# Efficacy and safety of oral branched-chain amino acid supplementation in patients undergoing interventions for hepatocellular carcinoma: a meta-analysis

**DOI:** 10.1186/s12937-015-0056-6

**Published:** 2015-07-09

**Authors:** Ling Chen, Yaqin Chen, Xiwei Wang, Hong Li, Hongmin Zhang, Jiaojiao Gong, Shasha Shen, Wenwei Yin, Huaidong Hu

**Affiliations:** Department of Clinical Nutrition, the Second Affiliated Hospital of Chongqing Medical University, 74 Linjiang Road,Central District, Chongqing, 400010 PR China

**Keywords:** Branched-chain amino acids, Hepatocellular carcinoma, Meta-analysis

## Abstract

Most hepatocellular carcinoma (HCC) patients have complications, including cirrhosis and malnutrition. The efficacy of dietary supplementation with oral branched-chain amino acids (BCAAs) in HCC patients undergoing interventions has not been confirmed. Relevant publications on the efficacy and safety of oral BCAA supplementation for HCC patients undergoing anti-HCC interventions through September, 2014 were searched for identification in the PubMed, Embase, Web of Science, and the Cochrane Library databases. The pooled risk ratio (RR) and standardized mean difference (SMD) were used to assess the supplementation effects. A total of 11 eligible studies (974 patients in total) were evaluated and included in our analysis. Oral BCAA supplementation helped to maintain liver reserve with higher serum albumin (SMD = 0.234, 95 % CI: 0.033–0.435, P = 0.022), and lower rates of ascites (RR = 0.545, 95 % CI: 0.316–0.938, P = 0.029) and edema (RR = 0.494, 95 % CI: 0.257–0.952, P = 0.035) than in the control group. BCAA supplementation seemed to be effective in improving mortality, especially in Child-Pugh class B patients, but the efficacy was not confirmed. Apparent effects were not found in improving HCC recurrence, total bilirubin, ALT, or AST. BCAA supplementation was relatively safe without serious adverse events. BCAA supplementation may be clinically applied in improving liver functional reserve for HCC patients and further improving the quality of life.

**Electronic supplementary material:**

The online version of this article (doi:10.1186/s12937-015-0056-6) contains supplementary material, which is available to authorized users.

## Introduction

Hepatocellular carcinoma (HCC) is one of the most common cancers and a major cause of mortality among cancer patients [1]. It is particularly common in east and southeast Asia [2]. Annually, approximately 500000 new patients are diagnosed with HCC around the world, while the disease causes an almost equivalent number of deaths. The primary reasons for the poor prognosis of HCC patients are its high rates of recurrence and resulting liver failure. Chronic infection with hepatitis B (HBV) or hepatitis C virus (HCV) is considered the most important risk factor in HCC tumorigenesis [3]. Other factors, such as excessive alcohol consumption, cigarette smoking, and environmental exposure to aflatoxin, also affect disease progression and response to treatment [4]. Thus, there is no single, effective treatment strategy which can be applied to all HCC patients.

Currently, several methods, including surgery, radio frequency ablation (RFA), percutaneous ethanol injection therapy, transcatheter arterial chemoembolization (TACE), hepatic arterial infusion chemotherapy (HAIC), radiation therapy and sorafenib, are used to treat HCC, depending on the physical condition of the patient [5, 6]. Liver transplantation is the ultimate treatment which, while achieving optimal results (a 5-year survival rate of 60–70 %), is a therapeutic strategy limited by the acute shortage of donor organs [7]. Apart from transplantation, however, all the treatment modalities currently deployed can cause damage to the liver as well a broader systemic damage. In many cases, HCC treatment is also complicated by the frequency of cirrhosis [8]. Underlying cirrhosis frequently leads to protein-calorie malnutrition commonly caused by poor appetite, cacochylia, compromised nutrient uptake, and disorders in protein synthesis [9, 10]. Liver damage caused by HCC treatment can aggravate malnutrition and can, on occasion, contribute to the development of ascites and edema, which are important factors in the prognosis of HCC [11, 12]. In addition, cancer patients often present with enhanced protein catabolism, which can in turn make malnutrition more acute [13].

As essential amino acids in humans, BCAAs (leucine, isoleucine, and valine) are not only components of proteins but also the source of glutamate, which detoxifies ammonia through glutamine synthesis in skeletal muscle [14]. In patients with hepatic encephalopathy, BCAA supplementation is currently recommended in the guidelines of the American Society for Parenteral and Enteral Nutrition and the European Society for Clinical Nutrition and Metabolism [15, 16]. In fact, recent experiments and clinical trials suggest the possibility of more expansive applications of BCAAs in liver disease. The malnutrition associated with cirrhosis has been shown to be alleviated by the administration of BCAAs in both rats and humans [17, 18]. Other studies published in recent years have emphasized the importance of nutritional BCAA supplementation in patients undergoing anti-HCC interventions such as surgery, RFA, HAIC or TACE [19–28]. However, studies have reported inconsistent results from BCAA supplementation for HCC patients even among patients receiving the same anti-HCC therapy, and thus the efficacy of oral BCAA supplementation for HCC patients is yet to be fully confirmed. Up to now, very few meta-analyses have been performed to verify this effectiveness; they were only studied on operative oral administration of BCAAs for patients with HCC or with small sample sizes, even after merging for effect values [29, 30].

The purpose of our meta-analysis is to survey currently available evidence and carry out a quantitative combination of relevant metrics to estimate the efficacy, as well as the safety, of BCAA supplementation in patients undergoing surgical and non-surgical interventions for HCC, as compared with patients receiving no BCAA supplementation.

## Methods

### Literature search

Potentially relevant studies regarding the efficacy of BCAAs on HCC were identified by searching the PubMed, Embase, Web of Science, and the Cochrane Libraries, using the key words “branched-chain amino acids,” “hepatocellular carcinoma,” and their abbreviations. Multiple synonyms were also used. The search was restricted to “human” and “English”. Reference lists of all retrieved documents were manually searched for potentially relevant reports missed by the intelligent retrieval systems mentioned above. The search was carried out in September 2014, and the whole selection process was completed independently by two investigators (LC and YQC). Inconsistent search results were resolved with the assistance of an arbiter (HDH), where necessary.

### Inclusion and exclusion criteria

Published studies comparing the efficacy of oral BCAA supplementation to no additional BCAA supplementation in HCC were used for our meta-analysis, if the study was eligible and the data available. Inclusion criteria were as follows: a) randomized controlled trials (RCTs), with retrospective and prospective cohort study designs, b) All patients were diagnosed with HCC and underwent therapies for HCC, whether surgical or non-surgical, c) Studies directly comparing additional oral BCAA supplementation to usual meals with no BCAA supplementation with a follow-up duration equal to or more than 24 weeks and d) All patients demonstrated good compliance in taking the supplements and were monitored by a dietician. Studies were excluded if they featured: a) non-comparative data or observational methodologies, b) No available outcome measures and a follow-up duration of less than 24 weeks, c) Patients with serious impairment of organ function due to respiratory, renal, or heart disease or d) Patients with serious hepatic disease but without HCC.

### Efficacy measures

The interesting outcome measures studied were as follows: a) Mortality and HCC recurrence were used as primary efficacy measures and b) Liver biochemical and clinical symbols were used as secondary efficacy measures. Data on mortality was studied at the time-points of 1 and 3 years, and HCC recurrence at the time-points of 1, 2, and 3 years. Liver biochemical data included serum albumin, total bilirubin, alanine aminotransferase (ALT), and aspartate aminotransferase (AST) at the time-points of 6 and 12 months. Clinical signs included instances of ascites and the occurrence of edema shortly after anti-HCC therapies. The side effects caused by BCAAs also received special attention.

### Data extraction

All data were independently extracted from the included studies by two investigators (LC and YQC), and, where possible, calculated and confirmed. Any dispute between investigators was resolved by discussion or by arbitration from an arbiter (HDH), when necessary. If useful data were presented indirectly by figures and graphs or through different metrics, they were translated into correlative patterns by using get-data software or relevant formulae. If mean values or standard deviation (SD) for analysis were unavailable, they were calculated from medians and ranges using relevant formulae [31].

### Study quality

The quality of all included RCTs was assessed using the revised Jadad quality scale, which graded the quality of a study from 0 (lowest) to 7 (highest) by examining randomization, blinding, allocation concealment, and drop-out. For cohort designs, the quality of studies was assessed by the Newcastle-Ottawa Scale (NOS) based on several standards including selection of cohorts, comparability of cohorts, and assessment of the outcomes.

### Statistical analysis

Data analysis was carried out with the use of Software Stata version 12.0 (Stata Corporation, College Station, TX, USA) and was based on an intent-to-treat principle. Both dichotomous and continuous variables were extracted in this analysis. For dichotomous outcomes, the results were presented as relative risk (RR) with a 95 % confidence interval (95 % CI), while continuous results were presented as standardized mean difference (SMD) with a 95 % confidence interval (95 % CI). The overall effects were measured using a z-score with a significance set at P < 0.05. An RR of approximately 1 (p > 0.05) indicated no statistical significance between BCAA and control groups while an RR value deviated from 1 (p < 0.05) represented statistical significance. A SMD of approximately 0 (p > 0.05) indicated no statistical significance between BCAA groups and control groups, while SMD values with a deviation from 0 (p < 0.05) were judged to be of statistical significance. Statistical heterogeneity was evaluated by using chi-square and I-square (I^2^) tests with a significance set at p < 0.1. P < 0.1 and I^2^ > 50 % were considered to be significant heterogeneity. The random-effect method was used to combine results if confirmed significant heterogeneity was observed; otherwise, the fixed-effect method was used. To assess sources of potential bias, sensitivity analyses were performed where required. Funnel plots were used to assess the publication bias of selected articles and any potential bias was judged by Begg’s test.

## Results

### Search results and study characteristics

**Fig. 1:**
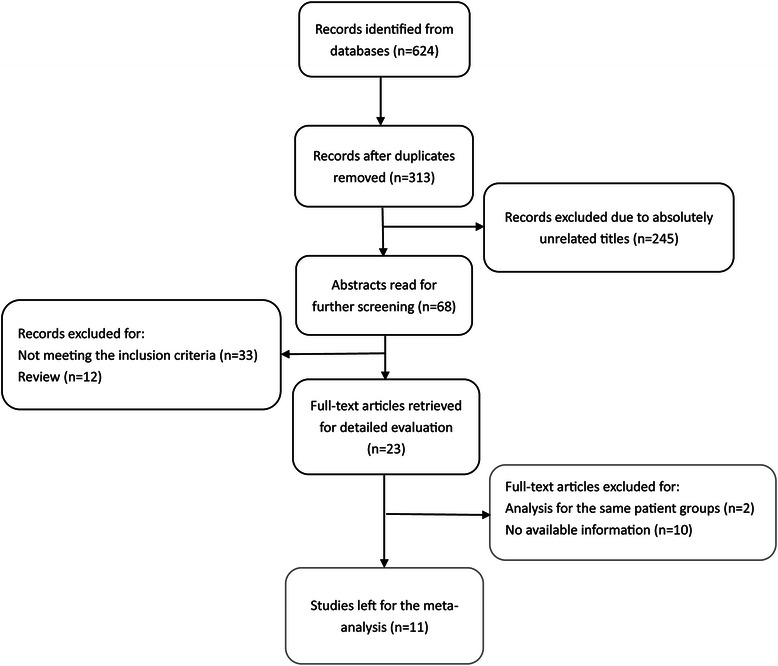
Flow diagram of literature selection process

As the search strategy shows (Fig. 1), 624 records in total were identified, and 311 records were removed as duplicate documents retrieved from two or more databases. The remaining studies received further screening by scanning the title or abstract, which resulted in the exclusion of a further 290 studies. As a result, 23 full-text articles were subjected to detailed evaluation, of which two were excluded because they analyzed the same patient groups, and a further ten were excluded due to lack of available data. Eventually, 11 eligible articles relating to a total of 974 subjects (450 in BCAA groups and 524 in control groups) were chosen for this meta-analysis. Of the 11 eligible studies, six were RCTs [19–24] and five were cohorts (of which three were retrospective and two prospective designs) [25–28, 32]. Out of the six RCTs, four received a Jadad score of 4 or 5 and were considered high-quality, while the other two were deemed low quality because of scores lower than 4 (Additional file 1: Table. S1). All five cohort studies received a NOS score of 7 or 8 (Additional file 2: Table S2). No publication bias was found among these included studies (Additional file 3: Figure S1). The detailed characteristics of the included studies are summarized in Table 1. All pooled results are summarized in Additional file 4: Table S3 and the results of each study reviewed are summarized in Additional file 5: Table S4.

**Table 1:** Characteristics of studies included in the meta-analysis

Study	Region	Group	N	Male/female	Age (year)	Child-Pugh grade: A/B/C	TMN stage: I/II/III/IV	Follow-up time (month)	Supplementation period (month)	Therapy for HCC	Study type
Nagasue et al.	Japan	BCAA	67	54/13	<50:5 50–70:55 > 70:7	53/13/1	24/35/7/1	35.8 ± 17.9	>12	surgery	RCT
1998		control	65	55/10	<50:7 50–70:45 > 70:13	50/14/1	22/36/6/1	36.0 ± 17.7			
Meng et al.	China	BCAA	21	19/2	51.5 ± 10.8	17/4/0	NA	17 (0.2–33)	3	surgery	RCT
1999		control	23	18/5	53.3 ± 12.8	20/3/0	NA	17 (2–33)			
Togo et al.	Japan	BCAA	21	17/4	66.5 ± 4.5	15/7/0	2/14/2/3	12	12	surgery	RCT
2005		control	22	17/5	64.3 ± 9.1	17/5/0	3/12/4/3	12			
Okabayashi et al.	Japan	BCAA	40	29/11	65.7 ± 8.6	33/7/0	NA	16 (2–47)	0.5	surgery	cohort
2008		control	72	55/17	68.3 ± 8.1	62/10/0	NA	23 (2–84)			
Ichikawa et al.	Japan	BCAA	26	18/8	64.7 ± 9.8	21/5/0	NA	40 (7–48)	6.5	surgery	RCT
2013		control	30	20/10	64.5 ± 11.4	25/5/0	NA	36 (6–50)			
Kuroda et al.	Japan	BCAA	20	13/7	65.6 ± 7.0	8/11/1	6/11/3/0	12	12	RFA	cohort
2010		control	15	9/6	66.0 ± 8.1	6/8/1	5/8/2/0	12			
Yoshiji et al.	Japan	BCAA	16	10/6	63.7 ± 10.8	12/4/0	10/5/1/0	48	48	RFA	RCT
2011		control	26	16/10	62.5 ± 11.5	21/5/0	18/7/1/0	48			
Nishikawa et al.	Japan	BCAA	115	64/51	69.3 ± 9.4	83/30/2	41/58/16/0	30 (2–95)	>1	RFA	cohort
2013		control	141	83/58	70.9 ± 7.8	88/52/1	60/60/21/0	29 (1–84)			
Poon et al.	China	BCAA	41	39/2	59 (24–84)	NA	30/11/0/0	29 (18–44 )	12.5	TACE	RCT
2004		control	43	39/4	59 (27–80)	NA	31/12/0/0	30 (18–43)			
Kanekawa et al.	Japan	BCAA	49	43/6	66.3 ± 7.0	23/26/0	0/0/41/8	NA	NA	HAIC	cohort
2014		control	43	34/9	68.0 ± 7.0	30/13/0	0/0/29/14	NA			
Takeda et al.	Japan	BCAA	33	27/7	72 (55–88)	16/18/0	0/2/15/17	NA	NA	sorafenib	cohort
2014		control	44	37/7	68 (46–89)	30/14/0	0/2/11/31	NA			

*RFA* radiofrequency ablation, *TACE*, transarterial chemoembolization, *HAIC*, hepatic arterial infusion chemotherapy; *RCT*, randomized controlled trial, *NA*, not available

### Mortality

**Fig. 2:**
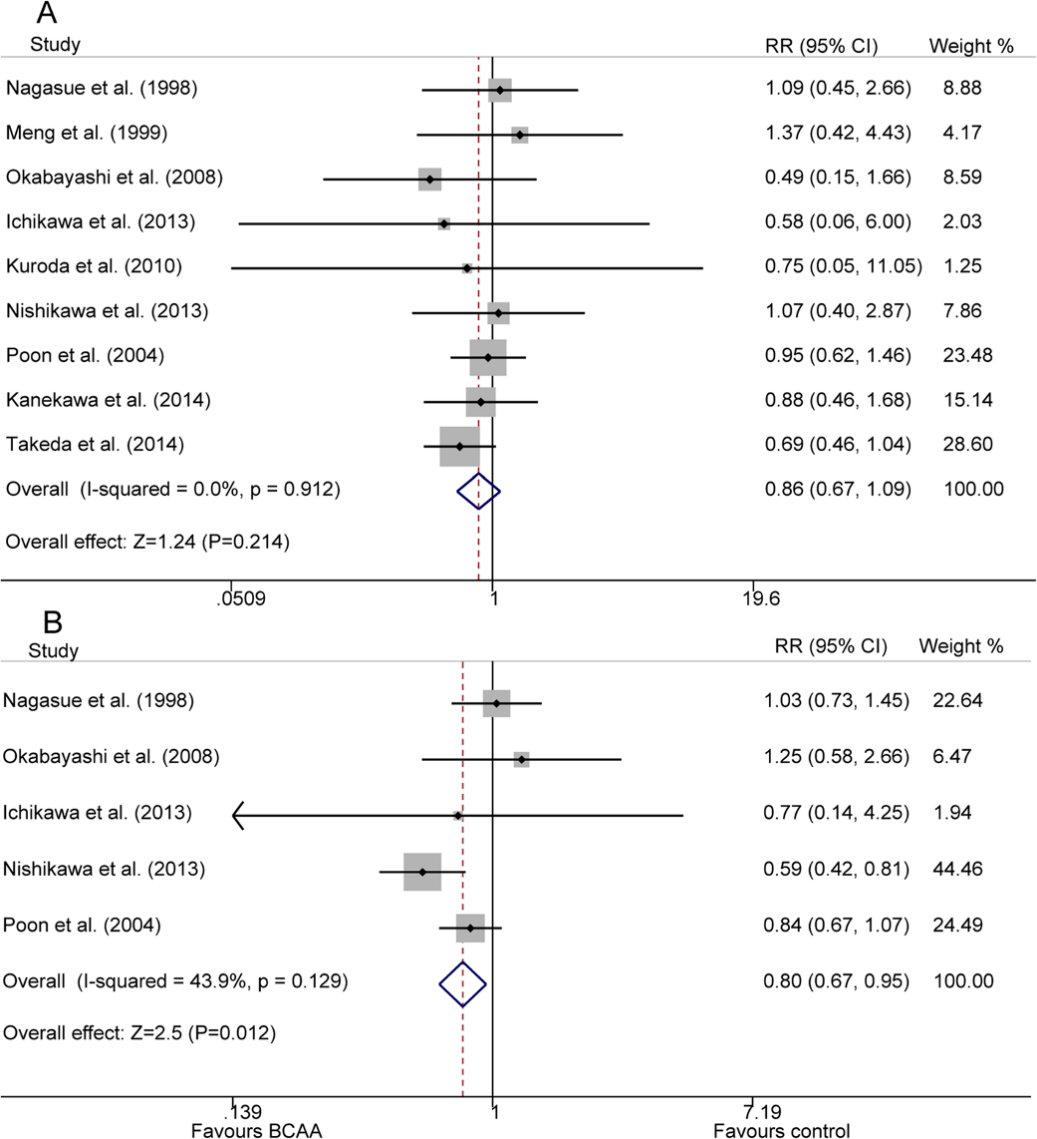
Forest map of summary estimates for comparison of mortality between BCAA and control groups. **a**) 1-year mortality; **b**) 3-year mortality

Information about 1-year mortality was reported in nine studies [19, 20, 22, 24–28, 32] while 3-year mortality was reported in five studies [19, 22, 24, 25, 27]. A fixed-effect model was used to analyze mortality at the two time-points since there was no significant heterogeneity among these studies (P = 0.912, I^2^ = 0.0 %; P = 0.129, I^2^ = 43.9 %, respectively). This meta-analysis showed that both 1-year and 3-year mortality were lower in BCAA groups when compared with control groups (18 % vs. 21 %; 37 % vs. 44 %, respectively). 1-year mortality did not achieve statistical significance (RR = 0.856, 95 % CI: 0.669–1.094, P = 0.214), while the difference became statistically significant with prolonged duration up to three years (RR = 0.797, 95 % CI: 0.667–0.952, P = 0.012) (Fig. 2).

Kanekawa, et al. [28] reported that the mortality of the BCAA group was significantly lower than that of control group for Child-Pugh class B patients, but there was no significant difference for Child-Pugh class A patients. Another three studies [26, 27, 32], which also reported significantly lower mortality in the BCAA groups compared with the control groups, were found to include a relatively higher proportion of Child-Pugh class B patients at baseline than that of studies [19, 20, 22, 25] which reported no significant difference in mortality (Table 2).

**Table 2:** The proportion of Child-Pugh class B patients at baseline in studies

Study	BCAA		Control		Total		
Nishikawa et al. 2013	30/115	26 %	52/141	37 %	82/256	32 %	**
Kanekawa et al. 2014	26/49	53 %	13/43	30 %	39/92	42 %	**
Takeda et al. 2014	18/33	55 %	14/44	32 %	32/77	42 %	**
Kuroda et al. 2010	11/20	55 %	8/15	53 %	19/35	54 %	**
Nagasue et al. 1998	13/67	19 %	14/65	22 %	27/132	20 %	*
Meng et al. 1999	4/21	19 %	3/24	13 %	7/45	16 %	*
Ichikawa et al. 2013	5/26	19 %	5/30	17 %	10/56	18 %	*
Okabayashi et al. 2008	7/40	18 %	10/72	14 %	17/112	15 %	*

**: Study that reported significantly lower mortality in the BCAA group compared with the control group

*: Study that reported no significant difference in mortality between the two groups

### HCC recurrence

The 1-year, 2-year, and 3-year recurrence of HCC were obtained in five [20–23, 26], three [20, 22, 23], and three [19, 22, 23] studies respectively. No significant heterogeneity was found at the three time-points (p = 0.940, I^2^ = 0.0 %; p = 0.308, I^2^ = 15.2 %; p = 0.948, I^2^ = 0.0 %, respectively). Similarly, a fixed-effect approach was used to estimate the overall effects. The rates of HCC recurrence were also lower in BCAA groups than control groups (11 % vs. 18 %; 30 % vs. 34 %; 54 % vs 56 %, respectively). However, at none of the three time-points did HCC recurrence achieve statistical significance (RR = 0.613, 95 % CI: 0.315–1.192, P = 0.149; RR = 0.882, 95 % CI: 0.544–1.431, P = 0.612; RR = 0.946, 95 % CI: 0.749–1.194, P = 0.638, respectively) (Additional file 6: Figure S2).

### Liver biochemical parameters

**Fig. 3:**
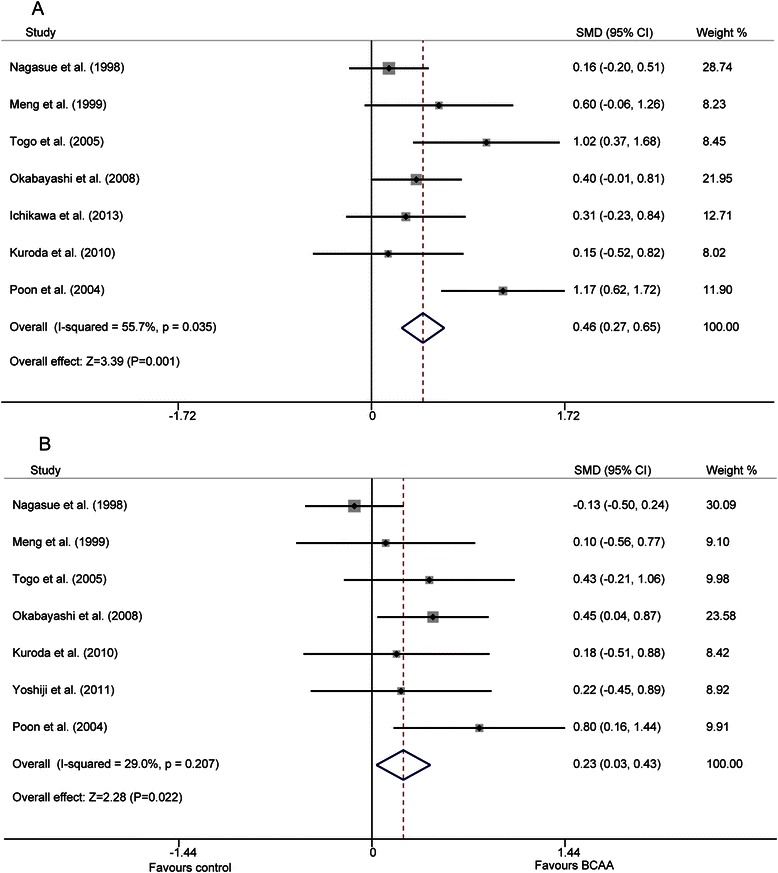
Forest map of summary estimates for comparison of serum albumin levels between BCAA and control groups. **a**) 6-month albumin; **b**) 12-month albumin

Liver biochemical parameters included serum levels of albumin, total bilirubin, ALT, and AST at the time-points of 6 and 12 months. The eligible articles showed no significant baseline difference among these parameters, so data at 6 and 12 months were suitable for direct comparison. Seven studies [19–22, 24–26] reported the 6-month albumin level and an equal amount of articles [19–21, 23–26] mentioned the 12-month albumin level. Significant heterogeneity was detected at 6 months (P = 0.035, I^2^ = 55.7 %) but not detected at 12 months (P = 0.207, I^2^ = 29.0 %). A random-effect model for 6 months and a fixed-effect model for 12 months showed that albumin levels were higher in both models for the BCAA groups compared with control groups (SMD = 0.515, 95 % CI: 0.217–0.812, P = 0.001; SMD = 0.234, 95 % CI: 0.033–0.435, P = 0.022, respectively) (Fig. 3). However, there were no significant differences between the two groups in terms of total bilirubin, ALT, and AST at 12 months (P = 0.454, P = 0.882, P = 0.235, respectively) on the basis of no significant heterogeneity among included studies of ALT and AST (P = 0.769, I^2^ = 0.0 %; P = 0.680, I^2^ = 0.0 %, respectively), but significant heterogeneity was found among the included studies in total bilirubin (p = 0.049, I^2^ = 58.0 %), which was analyzed using a random-effect model (Additional file 7: Figure S3).

### Clinical signs

**Fig. 4:**
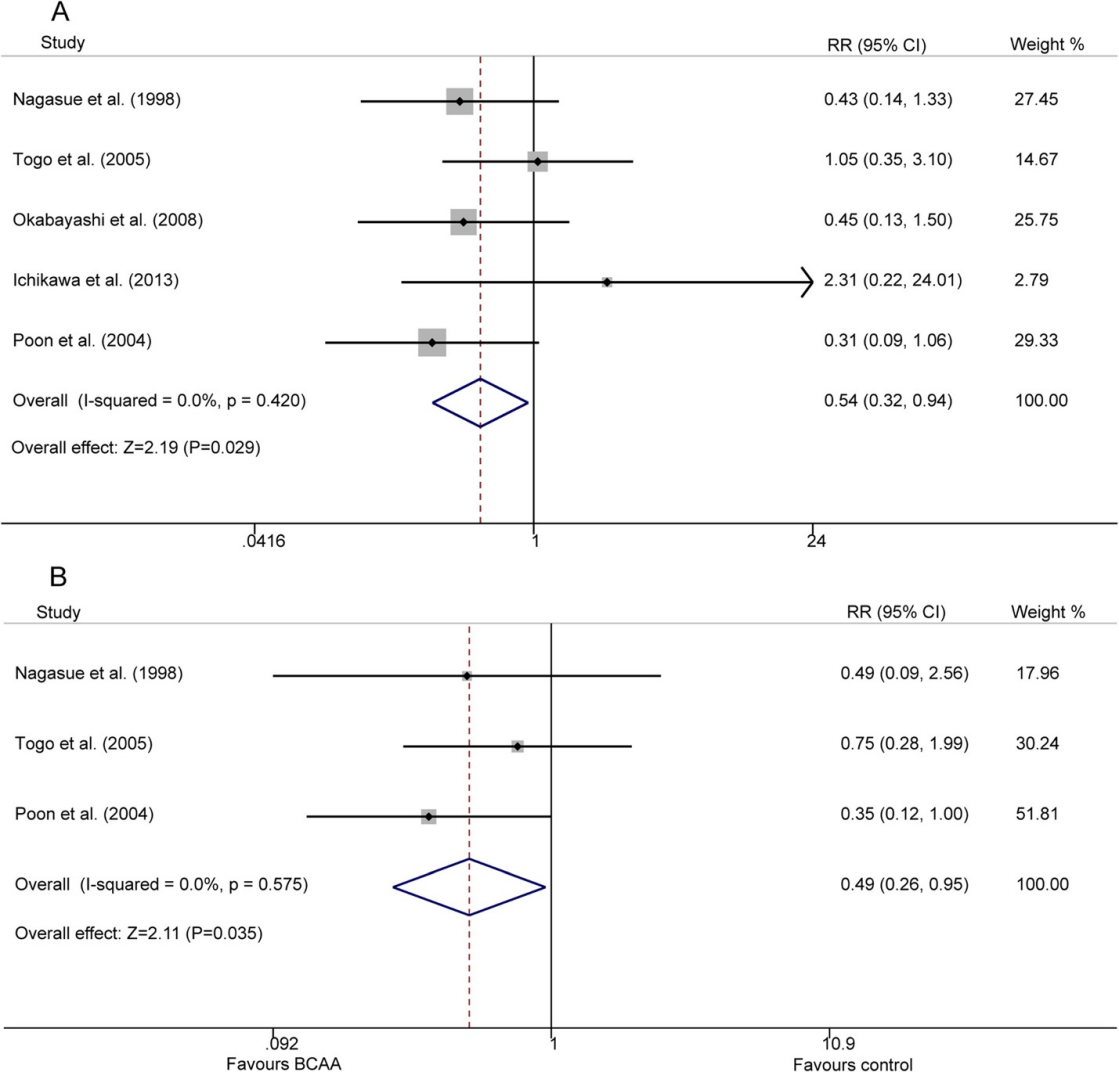
Forest map of summary estimates for comparison of ascites and edema between BCAA and control groups. **a**) ascites; **b**) edema

Five studies [19, 21, 22, 24, 25] provided data for ascites and three [19, 21, 24] reported the occurrences of edema a short time after therapies for HCC. No significant heterogeneity was found in ascites and edema (p = 0.420, I^2^ = 0.0 %; p = 0.575, I^2^ = 0.0 %, respectively). A fixed-effect analysis showed that the ascites rate in BCAA groups was distinctly lower when compared with control groups (9 % vs.16 %, P = 0.029), and a similar result was found in the edema rate (9 % vs. 16 %, P = 0.035) (Fig. 4).

### Side effects of BCAAs

Nagasue, et al. [19] reported that seven patients suffered side effects caused by BCAAs: nausea and vomiting in four, diarrhea in one, abdominal distension in one, and hypertension in one. Three of the reported patients could not continue BCAA supplementation. Meng, et al. [20] reported that two patients experienced occasional, diffuse abdominal pain after ingestion and one suffered from transient diarrhea, but all of these symptoms stopped spontaneously and none of the patients ended BCAA supplementation. Adverse reactions to BCAA supplementation were either not observed or not mentioned in the remaining articles. No serious events induced by BCAAs were reported in any of the included studies (Additional file 5: Table S4).

## Discussion

Most patients with HCC suffer from liver dysfunction associated with liver cirrhosis, tumor progression and tumor directed therapies. Because the liver is the central organ involved in the metabolism of nutrients, malnutrition occurs frequently when liver function is impaired [33–35]. The serum ratio of branched-chain amino acids (BCAAs) to aromatic amino acids (AAAs) stabilizes at a low level in liver cirrhosis and low serum BCAA/AAA ratio reduces biosynthesis and secretion of albumin in hepatocytes, which commonly leads to the occurrence of hypoproteinemia [36]. Hypoproteinemia or complications of hypoalbuminemia, such as ascites and edema, can increase the risk of discontinuing anti-cancer therapy [9]. BCAAs not only have a beneficial effect in meeting the increased energy requirements in cirrhotic patients, but also improve the level of albumin, thereby decreasing the rate of therapy interruption due to hypoproteinemia [37, 38]. In patients with diagnosed HCC who underwent interventions, optimal nutrition management may have benefits in terms of morbidity and mortality [22, 39].

In our meta-analysis, no statistical significance was found in the 1-year mortality between the BCAA and control groups (18 % vs. 21 %, P = 0.214). However, after expanding the follow up time, we found that 3-year mortality was statistically lower in the BCAA groups than in the control groups (37 % vs. 44 %, P = 0.012). One reason may be that, compared with patients analyzed at 1 year, patients analyzed at 3 years tended to take BCAA supplementation for a longer time. However, the BCAA supplementation period was just one of the many factors associated with mortality of HCC patients. Other factors, including age, sex, tumor stage, HCC recurrence, and Child-Pugh Classification also affect the mortality. Therefore, the efficacy of BCAA supplementation on improving 3-year mortality remains uncertain. Our results indicated that oral BCAA supplementation has more palpable effects on the mortality of Child-Pugh class B patients than that of Child-Pugh class A patients. A likely supposition is that Child-Pugh class B patients tend to have lower serum albumin levels but exhibit a stronger reaction to BCAA supplementation, with greater and more satisfactory recovery from previous hypoproteinemia, when compared with Child-Pugh class A patients. However, the underlying reasons for this phenomenon still need to be investigated and confirmed by further research.

Several human studies have demonstrated that insulin resistance (IR) is an HCC risk factor in patients with chronic hepatitis C and a similar close interaction was observed in patients with other morbidities, such as non-alcoholic fatty liver disease [40, 41]. BCAAs were found to suppress the incidence of hepatocarcinogenesis in patients with HCV-related cirrhosis and obesity that is often associated with IR through modulating insulin signaling [42]. The cumulative recurrence of HCC in patients with obvious insulin resistance was markedly inhibited by BCAA supplementation [43]. After merging effect values by meta-analysis, we found that the rates of recurrence, as the results section described above, were all lower in the BCAA groups, but with no statistical significance at any of the three time points (one, two, and three years). In fact, most of the included studies that mentioned information about HCC recurrence had small sample sizes, which may have affected our results through subordinate representation. On the other hand, the baseline information of IR in HCC patients in each included study is not clear, and the effects of BCAAs on HCC recurrence associated with IR could not be assessed in our present meta-analysis. This assessment should be conducted using larger and lengthier clinical trials.

Studies included in this meta-analysis showed a diversity of effects in improving liver function after BCAA supplementation (Additional file 5: Table S4). The results summarized in our analysis indicate a significantly higher level of serum albumin and significantly lower rates of ascites and edema in BCAA groups, but no significant differences were found in terms of total bilirubin, ALT, and AST. The higher serum albumin, lower ascites and reduced edema summarized in our data accord well with the clinical situation, because the most common reason for ascites and edema in HCC patients complicated by cirrhosis was hypoproteinemia, which can be greatly improved by supplementation with BCAAs.

Several limitations in our meta-analysis should be considered. First, some studies were not RCTs, of which three were retrospective cohort designs, and some reports with small sample sizes were also included. Secondly, the patients included were only from Asia, and most of these were Japanese. Thirdly, some data were not obtained directly; they were obtained through translation if the author did not respond to requests for detailed data, which may have affected the accuracy of our results. Finally, the studies were not identical in terms of the doses, types, or courses of supplementation. These differences in study design may explain the statistical heterogeneity.

In conclusion, patients receiving BCAA supplementation had a stronger functional liver reserve with higher serum albumin and lower rates of ascites and edema than those receiving no BCAA supplementation. However, the efficacy of BCAA supplementation on mortality, especially in Child-Pugh class B patients, is still not confirmed, even though the results of our meta-analysis demonstrated that oral BCAA supplementation was superior to the control group in improving 3-year mortality. There were no significant changes in HCC recurrence, total bilirubin, ALT, or AST. BCAA supplementation was relatively safe without serious adverse events for HCC patients. BCAA supplementation may be clinically applied to improve liver reserve of HCC patients and further improve their quality of life.

## Additional files

Additional file 1: Table S1. The quality of studies assessed by the revised Jadad scale. Additional file 2: Table S2. The quality of studies assessed by the NOS. Additional file 3: Figure S1. Funnel plots for some outcomes. A) 1-year mortality; B) 3-year mortality; C) 1-year HCC recurrence; D) 12-month serum albumin level. Additional file 4: Table S3. Results of meta-analysis comparing BCAA and control groups. Additional file 5: Table S4. The summary of results of each article included in our meta-analysis. Additional file 6: Figure S2. Forest map of summary estimates for rates of HCC recurrence in BCAA and control groups. A) 1-year recurrence; B) 2-year recurrence; C) 3-year recurrence. Additional file 7: Figure S3. Serum total bilirubin, ALT and AST levels 12 monthes after BCAA suplementation. A) Total bilirubin; B) ALT; C) AST.

## Abbreviations

HCC: Hepatocellular carcinoma
BCAAs: Branched-chain amino acids
RR: Risk ratio
SMD: Standardized mean difference
HBV: Hepatitis B virus
HCV: Hepatitis C virus
RFA: Radio frequency ablation
TACE: Transcatheter arterial chemoembolization
HAIC: Hepatic arterial infusion chemotherapy
AAAs: Aromatic amino acids
RCTs: Randomized controlled trials
ALT: Alanine aminotransferase
AST: Aspartate aminotransferase
SD: Standard deviation
NOS: Newcastle-Ottawa Scale
IR: Insulin resistance

## References

[1] Forner A, Llovet JM, Bruix J (2012). Hepatocellular carcinoma. Lancet.

[2] El-Serag HB, Rudolph KL (2007). Hepatocellular carcinoma: epidemiology and molecular carcinogenesis. Gastroenterology.

[3] El-Serag HB, Mason AC (2000). Risk factors for the rising rates of primary liver cancer in the United States. Arch Intern Med.

[4] Yu MC, Yuan JM (2004). Environmental factors and risk for hepatocellular carcinoma. Gastroenterology.

[5] Wilhelm SM, Carter C, Tang L, Wilkie D, McNabola A, Rong H, Chen C, Zhang X, Vincent P, McHugh M (2004). BAY 43–9006 exhibits broad spectrum oral antitumor activity and targets the RAF/MEK/ERK pathway and receptor tyrosine kinases involved in tumor progression and angiogenesis. Cancer Res.

[6] Cheng AL, Kang YK, Chen Z, Tsao CJ, Qin S, Kim JS, Luo R, Feng J, Ye S, Yang TS (2009). Efficacy and safety of sorafenib in patients in the Asia-Pacific region with advanced hepatocellular carcinoma: a phase III randomised, double-blind, placebo-controlled trial. Lancet Oncol.

[7] Llovet JM, Schwartz M, Mazzaferro V (2005). Resection and liver transplantation for hepatocellular carcinoma. Semin Liver Dis.

[8] Bruix J (1997). Treatment of hepatocellular carcinoma. Hepatology.

[9] Lautz HU, Selberg O, Korber J, Burger M, Muller MJ (1992). Protein-calorie malnutrition in liver cirrhosis. Clin Investig.

[10] Caregaro L, Alberino F, Amodio P, Merkel C, Bolognesi M, Angeli P, Gatta A (1996). Malnutrition in alcoholic and virus-related cirrhosis. Am J Clin Nutr.

[11] Bismuth H, Morino M, Sherlock D, Castaing D, Miglietta C, Cauquil P, Roche A (1992). Primary treatment of hepatocellular carcinoma by arterial chemoembolization. Am J Surg.

[12] Yamada R, Kishi K, Sonomura T, Tsuda M, Nomura S, Satoh M (1990). Transcatheter arterial embolization in unresectable hepatocellular carcinoma. Cardiovasc Intervent Radiol.

[13] O’Keefe SJ, Ogden J, Ramjee G, Rund J (1990). Contribution of elevated protein turnover and anorexia to cachexia in patients with hepatocellular carcinoma. Cancer Res.

[14] Yoshida T, Muto Y, Moriwaki H, Yamato M (1989). Effect of long-term oral supplementation with branched-chain amino acid granules on the prognosis of liver cirrhosis. Gastroenterol Jpn.

[15] Plauth M, Cabre E, Riggio O, Assis-Camilo M, Pirlich M, Kondrup J, Ferenci P, Holm E, Vom Dahl S, Dgem (2006). ESPEN guidelines on enteral nutrition: liver disease. Clin Nutr.

[16] ASPEN Board of Directors and the Clinical Guidelines Task Force. Guidelines for the Use of Parenteral and Enteral Nutrition in Adult and Pediatric Patients. Journal of Parenteral and Enteral Nutrition 2002, 26:1SA-138SA. 11841046

[17] Kajiwara K, Okuno M, Kobayashi T, Honma N, Maki T, Kato M, Ohnishi H, Muto Y, Moriwaki H (1998). Oral supplementation with branched-chain amino acids improves survival rate of rats with carbon tetrachloride-induced liver cirrhosis. Dig Dis Sci.

[18] Marchesini G, Zoli M, Dondi C, Bianchi G, Cirulli M, Pisi E (1982). Anticatabolic effect of branched-chain amino acid-enriched solutions in patients with liver cirrhosis. Hepatology.

[19] Mann DV (1998). Long-term oral administration of branched chain amino acids after curative resection of hepatocellular carcinoma: a prospective randomized trial. Br J Surg.

[20] Meng WC, Leung KL, Ho RL, Leung TW, Lau WY (1999). Prospective randomized control study on the effect of branched-chain amino acids in patients with liver resection for hepatocellular carcinoma. Aust N Z J Surg.

[21] Togo S, Tanaka K, Morioka D, Sugita M, Ueda M, Miura Y, Kubota T, Nagano Y, Matsuo K, Endo I (2005). Usefulness of granular BCAA after hepatectomy for liver cancer complicated with liver cirrhosis. Nutrition.

[22] Ichikawa K, Okabayashi T, Maeda H, Namikawa T, Iiyama T, Sugimoto T, Kobayashi M, Mimura T, Hanazaki K (2013). Oral supplementation of branched-chain amino acids reduces early recurrence after hepatic resection in patients with hepatocellular carcinoma: a prospective study. Surg Today.

[23] Yoshiji H, Noguchi R, Ikenaka Y, Kaji K, Aihara Y, Yamazaki M, Yamao J, Toyohara M, Mitoro A, Sawai M (2011). Combination of branched-chain amino acids and angiotensin-converting enzyme inhibitor suppresses the cumulative recurrence of hepatocellular carcinoma: a randomized control trial. Oncol Rep.

[24] Poon RT, Yu WC, Fan ST, Wong J (2004). Long-term oral branched chain amino acids in patients undergoing chemoembolization for hepatocellular carcinoma: a randomized trial. Aliment Pharmacol Ther.

[25] Okabayashi T, Nishimori I, Sugimoto T, Maeda H, Dabanaka K, Onishi S, Kobayashi M, Hanazaki K (2008). Effects of branched-chain amino acids-enriched nutrient support for patients undergoing liver resection for hepatocellular carcinoma. J Gastroenterol Hepatol.

[26] Kuroda H, Ushio A, Miyamoto Y, Sawara K, Oikawa K, Kasai K, Endo R, Takikawa Y, Kato A, Suzuki K (2010). Effects of branched-chain amino acid-enriched nutrient for patients with hepatocellular carcinoma following radiofrequency ablation: a one-year prospective trial. J Gastroenterol Hepatol.

[27] Nishikawa H, Osaki Y, Iguchi E, Koshikawa Y, Ako S, Inuzuka T, Takeda H, Nakajima J, Matsuda F, Sakamoto A (2013). The effect of long-term supplementation with branched-chain amino acid granules in patients with hepatitis C virus-related hepatocellular carcinoma after radiofrequency thermal ablation. J Clin Gastroenterol.

[28] Kanekawa T, Nagai H, Kanayama M, Sumino Y (2014). Importance of branched-chain amino acids in patients with liver cirrhosis and advanced hepatocellular carcinoma receiving hepatic arterial infusion chemotherapy. Cancer Chemother Pharmacol.

[29] Meng J, Zhong J, Zhang H, Zhong W, Huang Z, Jin Y, Xu J (2014). Pre-, peri-, and postoperative oral administration of branched-chain amino acids for primary liver cancer patients for hepatic resection: a systematic review. Nutr Cancer.

[30] Shu X, Kang K, Zhong J, Ji S, Zhang Y, Hu H, Zhang D (2014). Meta-analysis of branched chain amino acid-enriched nutrition to improve hepatic function in patients undergoing hepatic operation. Zhonghua Gan Zang Bing Za Zhi.

[31] Hozo SP, Djulbegovic B, Hozo I (2005). Estimating the mean and variance from the median, range, and the size of a sample. BMC Med Res Methodol.

[32] Takeda H, Nishikawa H, Iguchi E, Ohara Y, Sakamoto A, Saito S, Nishijima N, Nasu A, Komekado H, Kita R (2014). Effect of treatment with branched-chain amino acids during sorafenib therapy for unresectable hepatocellular carcinoma. Hepatol Res.

[33] Alberino F, Gatta A, Amodio P, Merkel C, Di Pascoli L, Boffo G, Caregaro L (2001). Nutrition and survival in patients with liver cirrhosis. Nutrition.

[34] Schutte K, Kipper M, Kahl S, Bornschein J, Gotze T, Adolf D, Arend J, Seidensticker R, Lippert H, Ricke J, Malfertheiner P (2013). Clinical characteristics and time trends in etiology of hepatocellular cancer in Germany. Digestion.

[35] Hsu WC, Tsai AC, Chan SC, Wang PM, Chung NN (2012). Mini-nutritional assessment predicts functional status and quality of life of patients with hepatocellular carcinoma in Taiwan. Nutr Cancer.

[36] Okuno M, Moriwaki H, Kato M, Muto Y, Kojima S (1995). Changes in the ratio of branched-chain to aromatic amino acids affect the secretion of albumin in cultured rat hepatocytes. Biochem Biophys Res Commun.

[37] Shinnick FL, Harper AE (1976). Branched-chain amino acid oxidation by isolated rat tissue preparations. Biochim Biophys Acta.

[38] Harper AE, Miller RH, Block KP (1984). Branched-chain amino acid metabolism. Annu Rev Nutr.

[39] Fan ST, Lo CM, Lai EC, Chu KM, Liu CL, Wong J (1994). Perioperative nutritional support in patients undergoing hepatectomy for hepatocellular carcinoma. N Engl J Med.

[40] Shintani Y, Fujie H, Miyoshi H, Tsutsumi T, Tsukamoto K, Kimura S, Moriya K, Koike K (2004). Hepatitis C virus infection and diabetes: direct involvement of the virus in the development of insulin resistance. Gastroenterology.

[41] Bugianesi E, McCullough AJ, Marchesini G (2005). Insulin resistance: a metabolic pathway to chronic liver disease. Hepatology.

[42] Muto Y, Sato S, Watanabe A, Moriwaki H, Suzuki K, Kato A, Kato M, Nakamura T, Higuchi K, Nishiguchi S (2006). Overweight and obesity increase the risk for liver cancer in patients with liver cirrhosis and long-term oral supplementation with branched-chain amino acid granules inhibits liver carcinogenesis in heavier patients with liver cirrhosis. Hepatol Res.

[43] Yoshiji H, Noguchi R, Namisaki T, Moriya K, Kitade M, Aihara Y, Douhara A, Yamao J, Fujimoto M, Toyohara M (2013). Branched-chain amino acids suppress the cumulative recurrence of hepatocellular carcinoma under conditions of insulin-resistance. Oncol Rep.

