# Supplementary material for: Efficacy and safety of oral branched-chain amino acid supplementation in patients undergoing interventions for hepatocellular carcinoma: a meta-analysis
Source: Nutr J. 2015 Jul 9;14:67. doi: 10.1186/s12937-015-0056-6 (PMC4496824; doi:10.1186/s12937-015-0056-6)
Supplement: Additional file 1: Table S1. — The quality of studies assessed by the revised Jadad scale. [file 12937_2015_56_MOESM1_ESM.docx]

**Table S1.** **Results of meta-analysis comparing BCAA and control groups.**

| Outcome of interest | | No. of studies | No. of patients | RR | 95% CI | p value | Heterogeneity | |
| --- | --- | --- | --- | --- | --- | --- | --- | --- |
|  |  |  |  |  |  |  | p value | I^2^ (%) |
| Mortality |  |  |  |  |  |  |  |  |
|  | 1-year | 9 | 889 | 0.856 | 0.669-1.094 | 0.214 | 0.912 | 0 |
|  | 3-year | 5 | 640 | 0.797 | 0.667-0.952 | 0.012 | 0.129 | 43.9 |
| HCC recurrence |  |  |  |  |  |  |  |  |
|  | 1-year | 5 | 220 | 0.613 | 0.315-1.192 | 0.149 | 0.94 | 0 |
|  | 2-year | 3 | 142 | 0.882 | 0.544-1.431 | 0.612 | 0.308 | 15.2 |
|  | 3-year | 3 | 230 | 0.946 | 0.749-1.194 | 0.638 | 0.948 | 0 |
| Ascites |  | 5 | 427 | 0.545 | 0.316-0.938 | 0.029 | 0.42 | 0 |
| Edema |  | 3 | 259 | 0.494 | 0.257-0.952 | 0.035 | 0.575 | 0 |
| Outcome of interest | | No. of studies | No. of patients | SMD | 95% CI | p value | Heterogeneity | |
|  |  |  |  |  |  |  | p value | I^2^ (%) |
| Albumin |  |  |  |  |  |  |  |  |
|  | 6-month | 7 | 453 | 0.515 | 0.217-0.812 | 0.001 | 0.035 | 55.7 |
|  | 12-month | 7 | 397 | 0.234 | 0.033-0.435 | 0.022 | 0.207 | 29 |
| Total bilirubin |  | 5 | 263 | -0.153 | -0.555-0.248 | 0.454 | 0.049 | 58 |
| ALT |  | 3 | 190 | -0.022 | -0.308-0.264 | 0.882 | 0.769 | 0 |
| AST |  | 3 | 189 | -0.174 | -0.460-0.113 | 0.235 | 0.68 | 0 |
