# Supplementary material for: Efficacy and safety of oral branched-chain amino acid supplementation in patients undergoing interventions for hepatocellular carcinoma: a meta-analysis
Source: Nutr J. 2015 Jul 9;14:67. doi: 10.1186/s12937-015-0056-6 (PMC4496824; doi:10.1186/s12937-015-0056-6)
Supplement: Additional file 2: Table S2. — The quality of studies assessed by the NOS. [file 12937_2015_56_MOESM2_ESM.docx]

**Table S2. The quality of studies assessed by the NOS.**

| Study | Selection | Comparability | Outcomes | NOS score |
| --- | --- | --- | --- | --- |
| Okabayashi et al. 2008 | 4 | 1 | 2 | 7 |
| Kuroda et al. 2010 | 4 | 1 | 2 | 7 |
| Nishikawa et al. 2013 | 4 | 1 | 3 | 8 |
| Kanekawa et al. 2014 | 4 | 1 | 3 | 8 |
| Takeda et al. 2014 | 4 | 1 | 3 | 8 |
