# Supplementary figures and images for: Efficacy and safety of oral branched-chain amino acid supplementation in patients undergoing interventions for hepatocellular carcinoma: a meta-analysis
Source: Nutr J. 2015 Jul 9;14:67. doi: 10.1186/s12937-015-0056-6 (PMC4496824; doi:10.1186/s12937-015-0056-6)

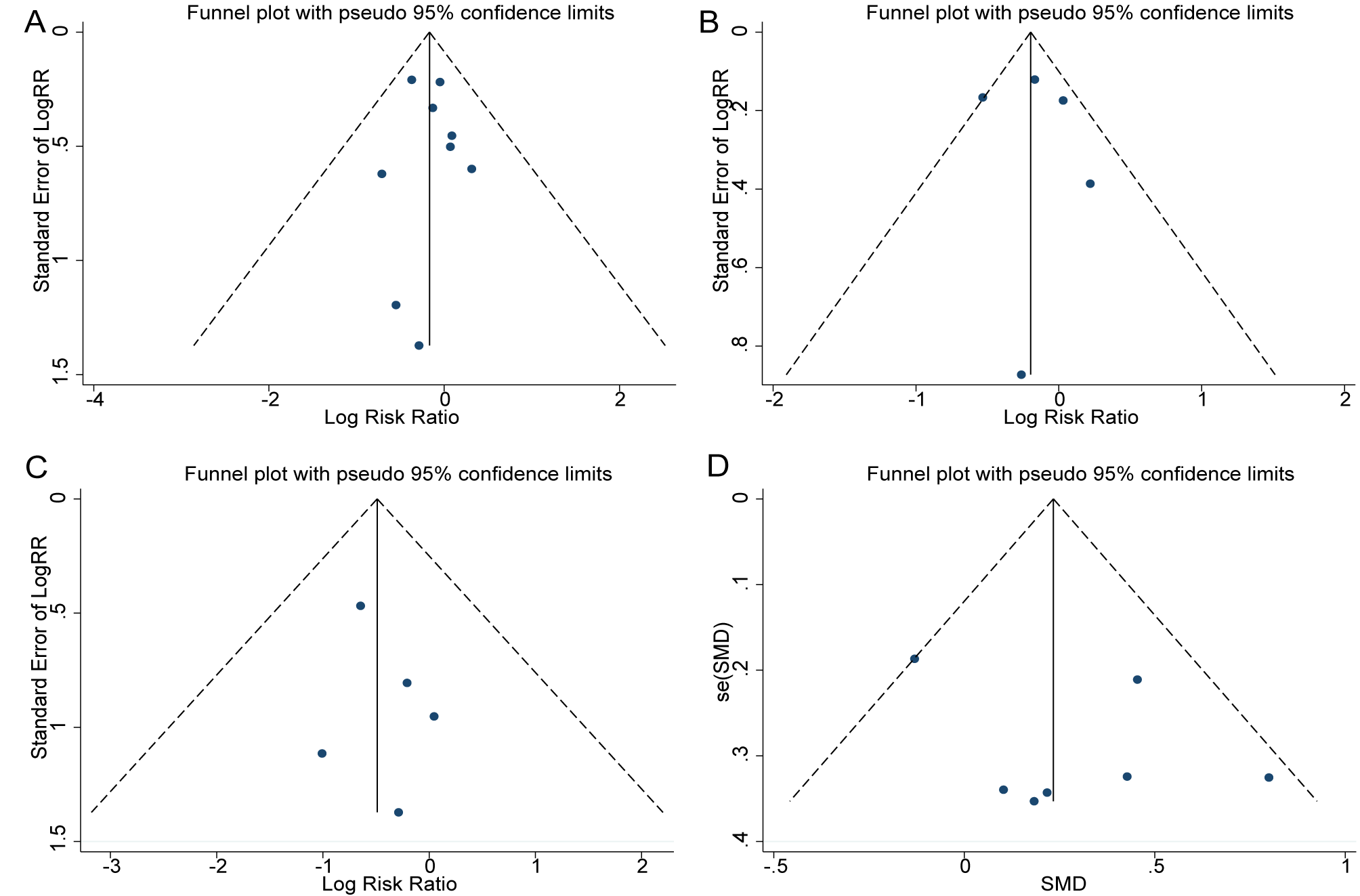

Supplement: Additional file 3: Figure S1. — Funnel plots for some outcomes. A) 1-year mortality; B) 3-year mortality; C) 1-year HCC recurrence; D) 12-month serum albumin level. [file 12937_2015_56_MOESM3_ESM.tiff]

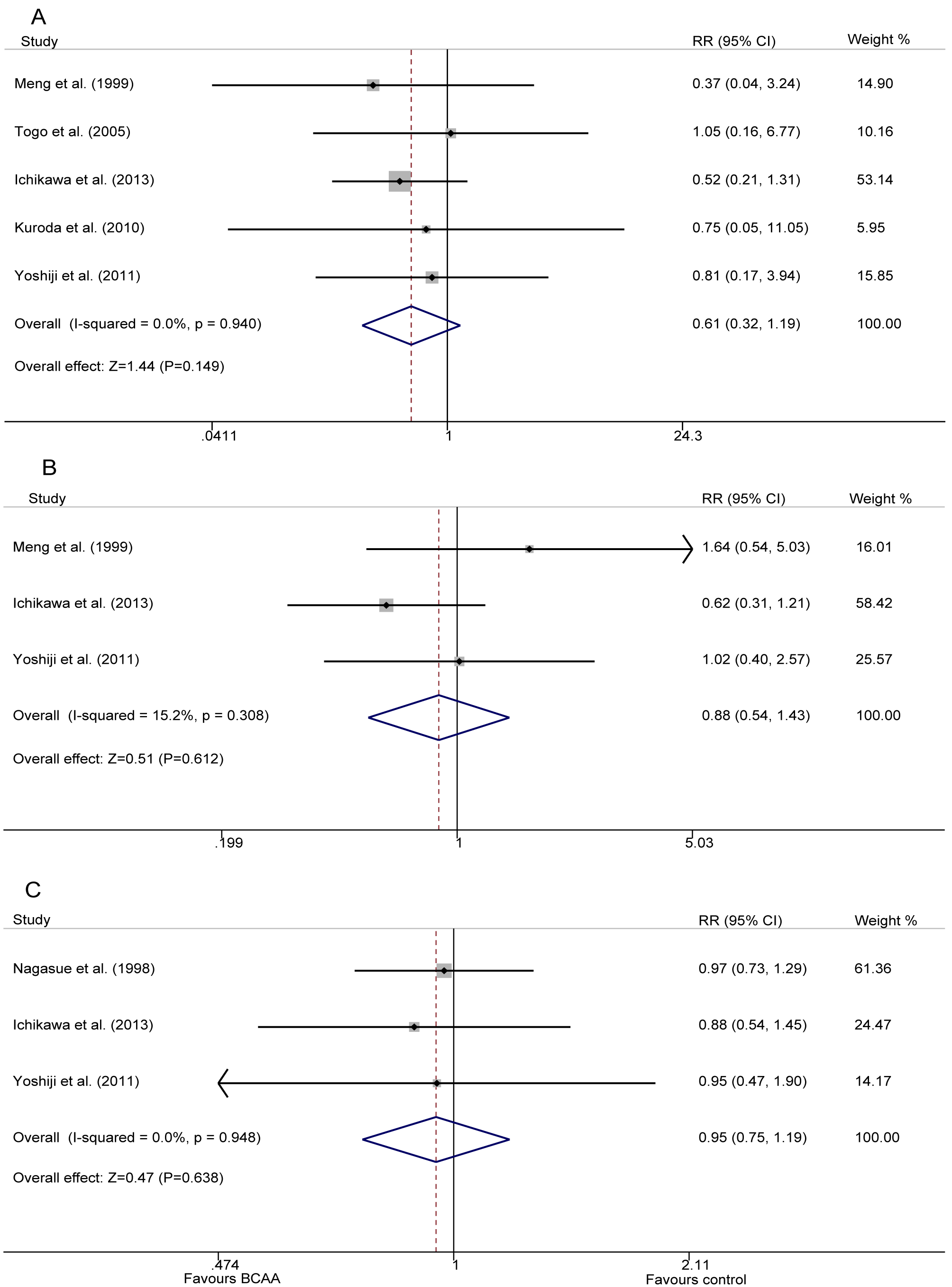

Supplement: Additional file 6: Figure S2. — Forest map of summary estimates for rates of HCC recurrence in BCAA and control groups. A) 1-year recurrence; B) 2-year recurrence; C) 3-year recurrence. [file 12937_2015_56_MOESM6_ESM.tiff]

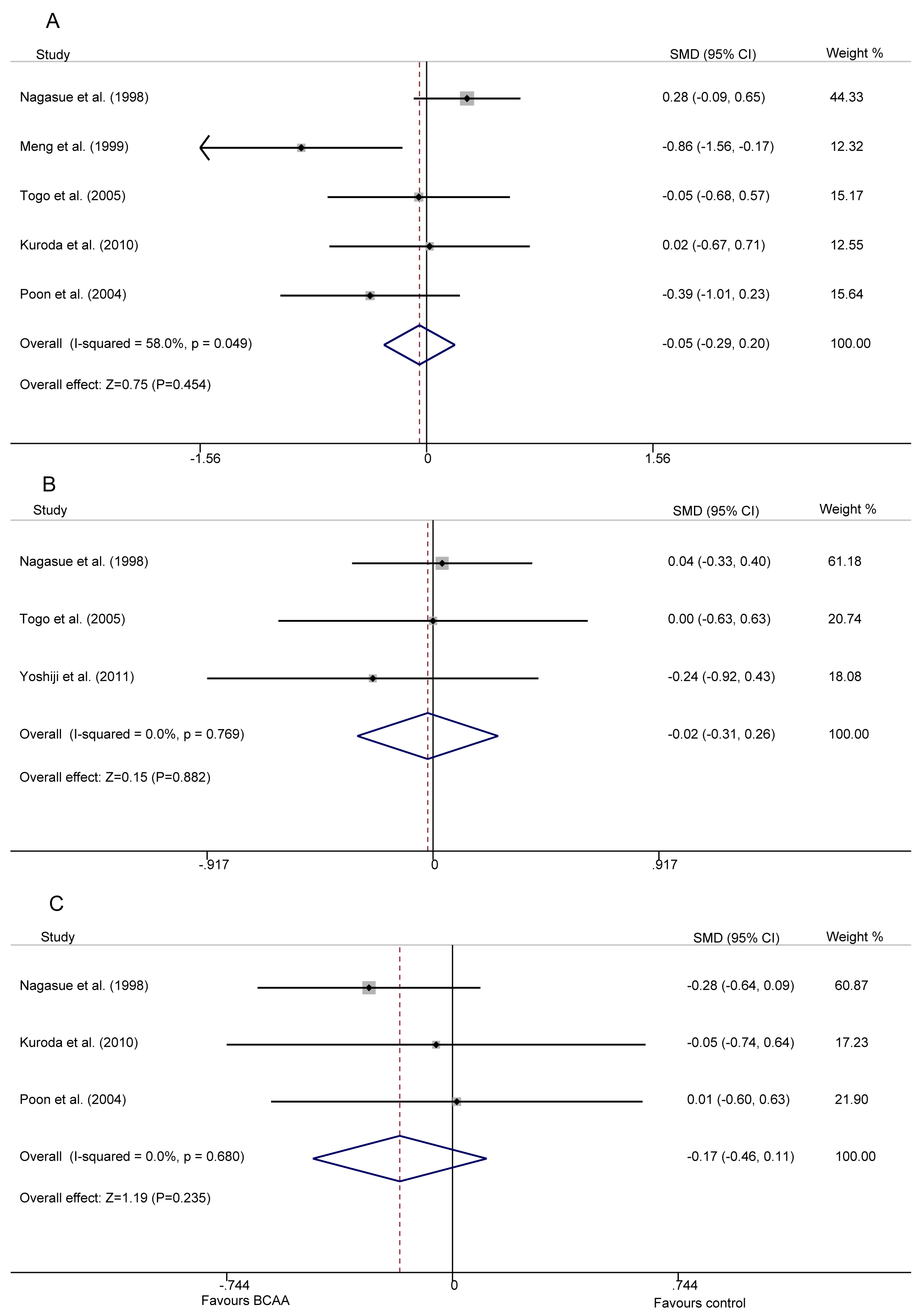

Supplement: Additional file 7: Figure S3. — Serum total bilirubin, ALT and AST levels 12 monthes after BCAA suplementation. A) Total bilirubin; B) ALT; C) AST. [file 12937_2015_56_MOESM7_ESM.tiff]
