# Supplementary material for: Efficacy and safety of oral branched-chain amino acid supplementation in patients undergoing interventions for hepatocellular carcinoma: a meta-analysis
Source: Nutr J. 2015 Jul 9;14:67. doi: 10.1186/s12937-015-0056-6 (PMC4496824; doi:10.1186/s12937-015-0056-6)
Supplement: Additional file 4: Table S3. — Results of meta-analysis comparing BCAA and control groups. [file 12937_2015_56_MOESM4_ESM.docx]

**Table S3. The quality of studies assessed by the revised Jadad scale.**

| Study | Randomization | Allocation concealment | Blinding | Dropout | Jadad score |
| --- | --- | --- | --- | --- | --- |
| Nagasue et al. 1998 | 1 | 0 | 0 | 1 | 2 |
| Meng et al. 1999 | 2 | 1 | 1 | 1 | 5 |
| Togo et al. 2005 | 2 | 1 | 0 | 1 | 4 |
| Ichikawa et al. 2013 | 1 | 0 | 0 | 1 | 2 |
| Yoshiji et al. 2011 | 1 | 2 | 0 | 1 | 4 |
| Poon et al. 2004 | 2 | 2 | 0 | 1 | 5 |
