# Supplementary material for: Efficacy and safety of oral branched-chain amino acid supplementation in patients undergoing interventions for hepatocellular carcinoma: a meta-analysis
Source: Nutr J. 2015 Jul 9;14:67. doi: 10.1186/s12937-015-0056-6 (PMC4496824; doi:10.1186/s12937-015-0056-6)
Supplement: Additional file 5: Table S4. — The summary of results of each article included in our meta-analysis. [file 12937_2015_56_MOESM5_ESM.docx]

**Table S4. The summary of results of each article included in our meta-analysis.**

| **Study** | **Supplementation period** | **Outcomes with improvement** | **Outcomes with no improvement** | **Side effects related to BCAA** |
| --- | --- | --- | --- | --- |
| Nagasue et al. (1998) | >12 months | Ascites, edema, albumin | Mortality, recurrence, ALT, AST, bilirubin | Nausea and vomiting in four; diarrhea in one; abdominal distension in one and hypertension in one |
| Meng et al. (1999) | 3 months | Albumin, bilirubin | Mortality, recurrence, ascites, edema | Occasional diffuse abdominal pain after ingestion in two and transient diarrhea in one |
| Togo et al. (2005) | 12 months | Ascites, edema, albumin | ALT, AST, bilirubin | No adverse reactions |
| Okabayashi et al. (2008) | 2 weeks | Ascites, albumin | Mortality | NA |
| Ichikawa et al. (2013) | 6.5 months | Recurrence, albumin | Mortality, ascites | No adverse reactions |
| Kuroda et al. (2010) | 12 months | Mortality, albumin | AST, bilirubin | NA |
| Yoshiji et al. (2011) | 48 months | a* | Recurrence, albumin, ALT | No adverse reactions |
| Nishikawa et al. (2013) | >1 month | Mortality, albumin | Recurrence | No adverse reactions |
| Poon et al. (2004) | 12.5 months | Ascites, edema, albumin, bilirubin, AST | Mortality | No adverse reactions |
| Kanekawa et al. (2014) | NA | Mortality, albumin | ALT, bilirubin | NA |
| Takeda et al. (2014) | NA | Mortality, albumin | b* | NA |

a*: no interesting outcomes reported with improvement; b*: no interesting outcomes reported with no improvement; NA: not available.
